# Low Cost, Easy to Prepare and Disposable Electrochemical Molecularly Imprinted Sensor for Diclofenac Detection

**DOI:** 10.3390/s21061975

**Published:** 2021-03-11

**Authors:** Isabel Seguro, João G. Pacheco, Cristina Delerue-Matos

**Affiliations:** REQUIMTE/LAQV, Instituto Superior de Engenharia do Porto, Instituto Politécnico do Porto, Rua Dr. António Bernardino de Almeida 431, 4200-072 Porto, Portugal; mgsps@isep.ipp.pt (I.S.); cmm@isep.ipp.pt (C.D.-M.)

**Keywords:** diclofenac, electrochemical sensor, molecularly imprinted polymer, disposable screen-printed electrode

## Abstract

In this work, a disposable electrochemical (voltammetric) molecularly imprinted polymer (MIP) sensor for the selective determination of diclofenac (DCF) was constructed. The proposed MIP-sensor permits fast (30 min) analysis, is cheap, easy to prepare and has the potential to be integrated with portable devices. Due to its simplicity and efficiency, surface imprinting by electropolymerization was used to prepare a MIP on a screen-printed carbon electrode (SPCE). MIP preparation was achieved by cyclic voltammetry (CV), using dopamine (DA) as a monomer in the presence of DCF. The differential pulse voltammetry (DPV) detection of DCF at MIP/SPCE and non-imprinted control sensors (NIP) showed an imprinting factor of 2.5. Several experimental preparation parameters were studied and optimized. CV and electrochemical impedance spectroscopy (EIS) experiments were performed to evaluate the electrode surface modifications. The MIP sensor showed adequate selectivity (in comparison with other drug molecules), intra-day repeatability of 7.5%, inter-day repeatability of 11.5%, a linear range between 0.1 and 10 μM (*r*^2^ = 0.9963) and a limit of detection (LOD) and quantification (LOQ) of 70 and 200 nM, respectively. Its applicability was successfully demonstrated by the determination of DCF in spiked water samples (river and tap water).

## 1. Introduction

All over the world, people benefit from thousands of synthetic chemicals that were developed in the last decades, improving populations’ health, aging and lifestyles. However, huge consumption rates and low waste treatment lead to a severe environmental issue [1]. Although essential for people’s well-being, pharmaceuticals products (PP) and their metabolites have become of major concern due to the potential impact on human health and the environment since they are very persistent and little biodegradable. The effluents from wastewater treatment plants (WWTP) may contribute to the release of PP since they are normally not designed for the removal of these compounds. The real impact of such contamination is not yet totally known, so a fast and cost-effective monitorization of PP in environmental samples is required [2].

Diclofenac (DCF) is a nonsteroid anti-inflammatory drug, with analgesic and antipyretic properties, widely used all over the world, both in human and in veterinary medicine. It is used in the treatment of rheumatic pain, joint inflammation [3] and can be administrated orally, topically or by intramuscular injection. 75% of DCF consumed enters the water cycle [4], and due to poor degradation, hydrophilicity, stability [5] and higher consumption rates, this drug is frequently detected in rivers [6], sediments, sludges [7] and even in drinking water [8,9]. Abnormalities associated with DCF exposure are reported in specific ecosystems, namely in the vulture population in India [7], aquatic species [10,11] and plants [9]. The most common method for DCF analysis in environmental samples, with high sensitivity and low limits of detection, is liquid chromatography (LC) [4,12,13]. However, LC methods are time-consuming, laborious, expensive in equipment, solvents and working hours.

Therefore, there is a demand for the development of simple, low-cost, environmentally friendly analytical methods for contaminants determination. It is also important that these methods can be used on large-scale and as portable and miniaturized tools. Electrochemical sensors have been showing their ability to address some of those characteristics’ specialty due to the high sensitivity, fast response, small size of equipment, easy installation, simple sample preparation and suitability for in situ analysis [14,15,16]. There are several reports on the electrochemical detection of DCF [17,18,19,20]. Although the performance and sensitivity of these methods were quite good, they are not selective, and in most cases, the preparation or application in real situations is very difficult. The coupling of sensing elements with electrochemical detection to prepare highly selective sensors has been a scientific area of substantial development and growth over the last 20 years. Among several strategies, the combination of molecularly imprinted polymers (MIPs) as sensing elements in electrochemical analysis in the last years has proved to be one of the most promising techniques [21,22,23,24,25].

Bulk (3-D) polymerization is the most common procedure to obtain MIPs. It starts with the polymerization of selected functional monomers (either organic or inorganic materials) around a target analyte (template) in the presence of a crosslinker agent and a solvent. Then, the entrapped template molecule is removed, typically by solvent extraction and a polymer matrix, with sites complementary in shape, size, and functionality to the imprinted molecule is produced [26,27]. The obtained polymer shows specific binding sites with a high affinity to the imprinted molecule compared to other molecules [27]. The advantages of using MIPs, compared with biological systems, such as enzymes and antibody/antigen, are that they are robust, more stable to chemical and thermal conditions, easy to prepare, reusable and low-cost.

One of the main challenges in MIP electrochemical preparation is the modification of the transducer (electrode) with the polymer particles. Surface imprinting (2-D) directly on the electrode surface by electropolymerization proved to be an interesting and efficient choice. It is based on the use of monomers that polymerize by the passage of electric currents, such as pyrrole, o-phenylenediamine, aniline and 4-aminobenzoic acid. The process is much faster than traditional bulk polymerization. It is very simple and permits easy control of the polymer thickness and morphology by adjusting the electrochemical conditions. Moreover, it enables direct communication between the coating and the surface of a transducer [28,29]. Several examples of this type of sensor can be found, such as drugs [30,31], explosives [32], biomarkers [33,34] and pesticides [35].

In the present study, MIP technology was used to construct a voltammetric sensor for the analysis of DCF. The MIP sensor was produced by electropolymerization of dopamine (DA) on the surface of a screen-printed carbon electrode (SPCE). DA shows goods properties to be electropolymerized and used in MIPs preparation [36,37,38,39]. The aim of this work was to aid the development of selective, low-cost, easy to handle and disposable methods for DCF determination. The proposed sensor can be easily prepared and managed. After optimization and analytical validation, it was applied to the analyses of river and tap water. MIPs electrochemical [40,41] and optical sensors [42] for DCF were already proposed. However, to the best of the authors’ knowledge, none was developed as a disposable device with the ability to integrate portable platforms.

## 2. Materials and Methods

### 2.1. Reagents

Commercial reagents were of analytical grade and used without purification. Dopamine hydrochloride and diclofenac sodium were obtained from Sigma-Aldrich. DCF stock solution was prepared by weighing the appropriate amount of the drug and dissolving it in methanol from ROMIL pure chemistry. Working solutions were prepared daily in 0.1 M HCl from Fluka. Potassium chloride was obtained from VWR chemicals, sodium hydroxide and sulfuric acid, both from Merck. Mefenamic acid (MFA), carbamazepine (CBZ), acetylsalicylic acid (ASA) and amitriptyline (AMI) were all obtained from Sigma-Aldrich. Ultra-pure water (resistivity = 18.2 MΩ∙cm) obtained from a Millipore (Simplicity 185) water purification system was utilized in all experiments. Phosphate buffer solution (0.1 M, pH = 7) was prepared with KH_2_PO_4_ and K_2_HPO_4_ (Riedel-de-Haën).

### 2.2. Apparatus

The electrochemical experiments, such as CV and DPV, were carried out with an Autolab PGSTAT 101 potentiostat-galvanostat controlled by Nova 1.10 software (Metrohm Autolab). For EIS analysis, an Autolab PGSTAT 128 N potentiostat-galvanostat with NOVA 1.6 software was used (Metrohm Autolab). In this study, commercial screen-printed carbon electrodes (SPCE, Dropsens, DRP-110) with a carbon working electrode (d = 4.0 mm), carbon auxiliary electrodes and a silver pseudo-reference electrode were used.

### 2.3. MIP Sensor Fabrication

A bare SPCE was initially activated in 0.5 M H_2_SO_4_ by CV between 0.0 V and 1.2 V with a scan rate of 100 mV/s for 10 cycles. The MIP sensor was then obtained by electropolymerization using CV. 40 µL of a polymerization solution with 20 mM DA and 5 mM DCF in KCl 0.1 M was placed on the SPCE working electrode, and CV was performed from −0.5 V to 1.0 V at 100 mV/s during 10 cycles. A NIP was prepared under the same conditions, without DCF. After polymerization, DCF entrapped molecules were removed with a methanol/NaOH 0.1 M (50:50, *v*/*v*) solution placed on the SPCE and regenerated each 20 min for 2 h, producing the selective cavities in the MIP.

### 2.4. Electrochemical Measurements

All measurements were performed at room temperature. The determination of DCF was performed by DPV. 10 µL of a DCF solution was placed at the sensor for 30 min for DCF rebind. Then the sensor was washed with water and left to dry. Finally, 40 µL of PBS 0.1 M pH = 7 was used as support electrolyte for DCF analysis by DPV between 0.4 V and 1.0 V. The scan rate was 50 mV/s with a step potential of 5 mV. CV and EIS characterization were performed with 40 µL of 0.5 mM of [Fe(CN)_6_]^3−/4−^ in 0.1 M KCl. EIS was conducted using a sinusoidal signal of amplitude 10 mV, with a frequency range from 10 mHz to 0.1 MHz and the fixed electrical potential of 0.2 V and CV with a scan rate of 100 mV/s, from −0.2 to +0.6 V.

### 2.5. Sample Analysis

Tap water and river water from Lis river, Portugal, were tested. The samples were spiked with DCF and then analyzed without further treatment under the conditions described above.

## 3. Results and Discussion

### 3.1. Molecularly Imprinting of DCF at SPCE

**Figure 1:**
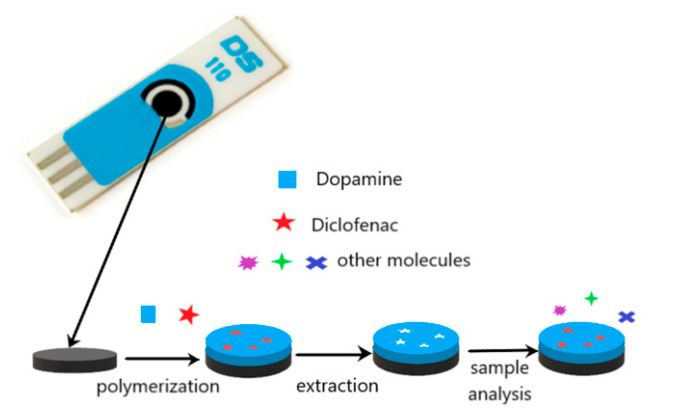
Schematic illustration of the preparation of the molecularly imprinted polymer (MIP)/screen-printed carbon electrode (SPCE) sensor.

The preparation of a selective sensor for DCF was carried out by electropolymerization on the surface of the carbon working electrode. This approach was chosen due to its simplicity, low-cost and simultaneous polymerization and attachment to the electrode. Figure 1 illustrates the construction of the sensor.

**Figure 2:**
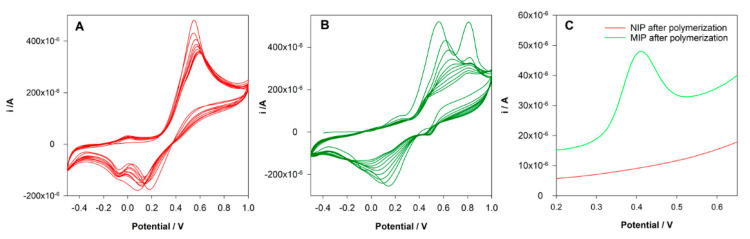
(**A**) Electropolymerization of non-imprinted control sensors (NIP) from a solution containing 20 mM 0.1 M HCl; (**B**) electropolymerization of MIP from a solution containing 20 mM dopamine (DA) and 5 mM diclofenac (DCF) in 0.1 M H; (**C**) differential pulse voltammetry (DPV) analysis of NIP and MIP sensors after rebinding to DCF.

This type of polymerization can be easily performed in aqueous solutions and is a good choice for compounds that show good solubility in aqueous solutions as DCF. Among several monomers tested, DA was selected. A non-covalent approach was used for MIP preparation. A pre-polymerization solution with 5 mM DCF and 20 mM DA in KCl 0.1 M was prepared and left in contact for 10 min. Then 40 µL of the solution was placed on the surface of the SPCE, and cyclic voltammetry was conducted in the range between −0.5 and 1.0 V at a scan rate of 100 mV/s for 10 cycles. The same procedure was followed to prepare a control non-imprinted electrode (NIP) but in the absence of DCF in the polymerization solution. Figure 2A shows the electrochemical polymerization of DA. It is possible to observe the formation and growth of the poly-DA film layer-by-layer at the surface of the electrode. An oxidation peak of DA at 0.6 V was observed during the polymerization, and two reduction peaks were registered at 0.2 and −0.1 V. The intensity of the peak’s current decreases along with the polymerization without a total block of the current, indicating the formation of a conductive polymer. During the formation of the MIP, differences were found (Figure 2B). The oxidation peak of DA was at 0.5 V, and an oxidation peak of DCF was registered at 0.8 V. The decrease of the DA peak during the polymerization was more pronounced. Instead of two reduction peaks, a single and large reduction peak was observed. These differences can be explained by the interaction between DA and DCF and the entrapment of the DCF molecules in the polymeric matrix.

In order to evaluate the success of the imprinting process, the electrodes were washed with water, and a DPV analysis in PBS 0.1 M pH = 7 was performed. Figure 2C shows the obtained voltammograms. As expected, it was not registered any peak at NIP/SPCE. However, an oxidation peak at 0.4 V was registered at MIP/SPCE, which can be attributed to the oxidation of imprinted DCF molecules, proving the imprinting in the polymer.

### 3.2. Electrochemical Behavior of DCF at MIP/SPCE

**Figure 3:**
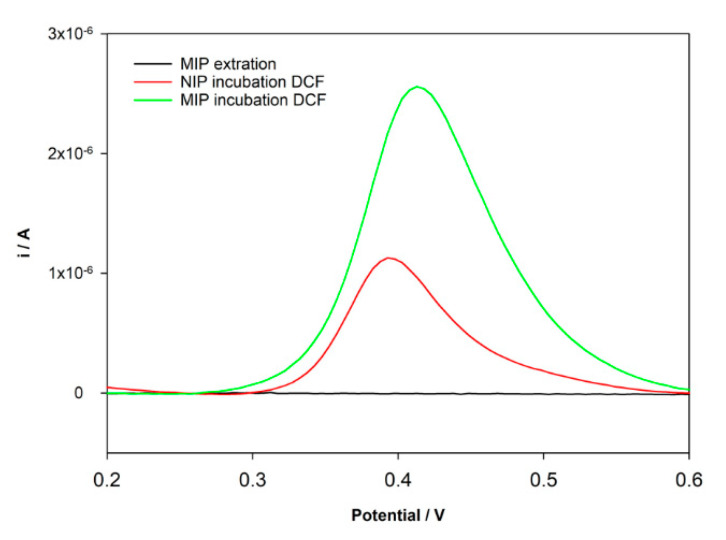
DPV voltammograms after incubation DCF solution.

After MIP preparation, the extraction and the ability to rebind to the template molecule should be assured. The extraction of the imprinted template is a critical and fundamental step and one of the major difficulties in MIPs preparation. This step is essential to create the cavities highly selective to the template. In the case of MIP electrochemical sensors, it is important to assure that no electrochemical peak appears after extraction. A Methanol/NaOH 0.1 M (50:50, *v*/*v*) solution was used to remove the DCF molecules entrapped in the MIP structure. As intended, the oxidation peak of DCF does not appear after extraction (Figure 3). The ability to rebind DCF molecules at the newly constructed sensor was tested after the incubation of a 10 µM DCF standard solution prepared in HCl for 30 min in both NIP/SPCE and MIP/SPCE. The results for DPV analysis in PBS pH = 7 are shown in Figure 3. It can be seen that an oxidation peak of DCF at 0.4 V was registered for both NIP/SPCE and MIP/SPCE after rebinding; however, for MIP/SPCE, the analytical signal is substantially higher. The peak presented in the NIP/SPCE may be explained by nonspecific binding between DCF and poly-DA. Otherwise, the higher oxidation peak at MIP/SPCE can be explained by the formation of specific binding sites provided by the successful imprinting process witch result in a higher electron transference. Using these results, an imprinting factor (ip current MIP/ip current NIP) of 2.5 was estimated.

### 3.3. Optimization of Experimental Conditions

The final performance of the sensor depends on several important parameters, which were investigated and optimized by DPV. These include polymerization conditions, such as the concentration of monomer (DA), the concentration of template (DCF) and the number of polymerization cycles. After preparation, the extraction and incubation conditions are also important parameters to study.

#### 3.3.1. Polymerization Conditions

Initially, the influence of the monomer concentration was studied by preparing several sensors with different DA concentrations (1, 5, 10, 20, 40 mM).

**Figure 4:**
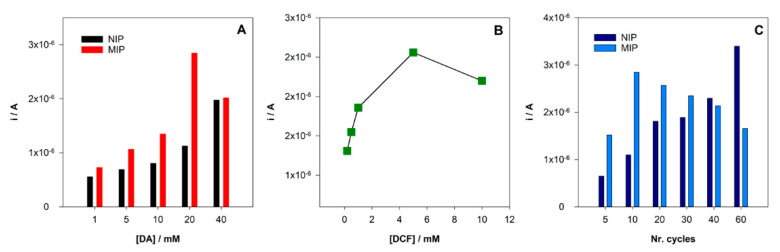
Optimization of the MIP preparation conditions: (**A**) variation of the peak current intensity with the concentration of monomer DA; (**B**) variation of the peak current intensity with the concentration of template DCF; (**C**) variation of the peak current intensity with a number of CV cycles.

As expressed in Figure 4A, for MIP/SPCE, the signal increase with increasing concentrations of the monomer (DA) up to 20 mM and then a decrease in current response was observed for 40 mM of DA. On the other hand, the NIP/SPCE signal increases over the range of concentrations tested. An explanation for this behavior may be related to the increase of conductivity of the electrode surfaces with the formation of more poly-DA. However, in the MIP/SPCE, the higher increase in the concentration of the monomer could lead to more entrapment of DCF and more difficulties in the extraction from cavities formation along with more trouble of DCF to diffuse through the polymer matrix. The higher difference in the peak current between MIP/SPCE and NIP/SPCE was achieved for 20 mM DA concentration, so it was chosen for the MIP construction.

After choosing the monomer concentration with the higher peak current, the better template concentration was also explored. Keeping all other analysis conditions the same, the DCF concentration was changed from 1 to 10 mM. It was found an increase of the peak current (Figure 4B) until 5 mM and then a decrease when using 10 mM. Again, if the DCF concentration is too high during the polymerization, it could be too entrapped in the polymer. Therefore, 5 mM of DCF was chosen for subsequent analysis.

The number of polymerization cycles is an important parameter when using electropolymerization because it can control the polymer film thickness formed at the surface of the electrode. Hence, polymerization cycles between 5 and 60 were tested. In NIP/SPCE, the peak current intensity increases with the number of scans. Conversely, for MIP/SPCE, the analytical signal is lower for 5 scans, increases for 10 and then constantly decreases until 60 cycles. On one hand, more polymerization cycles can increase the conductivity of the electrode. On the other hand, they increase the thickness of the film, making the extraction of the DCF molecules more difficult. Using 10 cycles in polymerization is achieved the higher peak current intensity for MIP/SPCE and the larger difference between MIP/SPCE and NIP/SPCE response, so for this number of scans, there is better sensitivity and selectivity.

#### 3.3.2. Incubation Time

**Figure 5:**
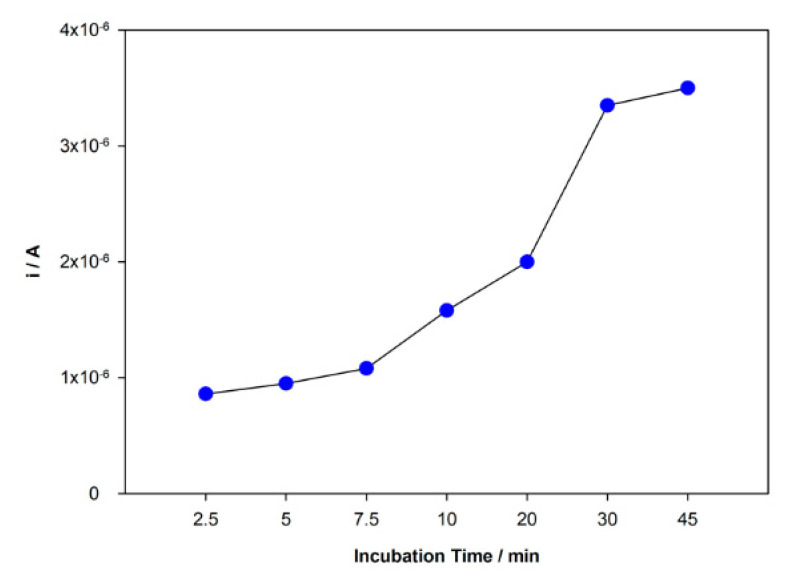
Variation of the peak current intensity with incubation time.

The rebind and adsorption of DCF molecules at the surface of the sensor are directly related to the incubation time. This parameter was studied in the range time from 2.5 to 45 min, and the peak current responses were recorded (Figure 5). An increasing peak current intensity with time was observed in this range until 30 min and then stabilization of the increase. Although the response was slightly higher for 45 min, the difference between 30 and 45 min was not significant enough to justify a longer incubation period. Hence, 30 min was chosen as incubation time.

#### 3.3.3. Extraction Conditions

The extraction of the template molecules entrapped in the polymeric matrix is crucial in MIPs preparation since it will produce the cavities with selective binding sites towards the imprinted template. In this work, several solvents were selected to extract DCF molecules from the poly-DA film obtained, including 0.1 M phosphate buffer at pH = 7, MeOH, 0.1 M HCl, 0.1 M HCl/MeOH (50:50, *v*/*v*) mixture and 0.1 M NaOH/MeOH (50:50, *v*/*v*). The extraction was considered complete when no oxidation peak of DCF was registered in DPV analysis. After extraction with the tested solvents, the incubations and DPV analysis of DCF was performed. It was found that a better response was obtained using 0.1 M NaOH/MeOH (50:50, *v*/*v*) as the extraction solvent and 2 h as the extraction time.

### 3.4. CV and EIS Characterization

CV and EIS analysis were performed using a solution of 0.5 mM [Fe(CN)_6_]^3−/4−^ in 0.1 M KCl solution after the different modifications of the SPCE electrode. The goal of this study was to show the differences at the surface of the electrodes, namely between the NIP and MIP films, proving that polymers with different characteristics were obtained.

**Figure 6:**
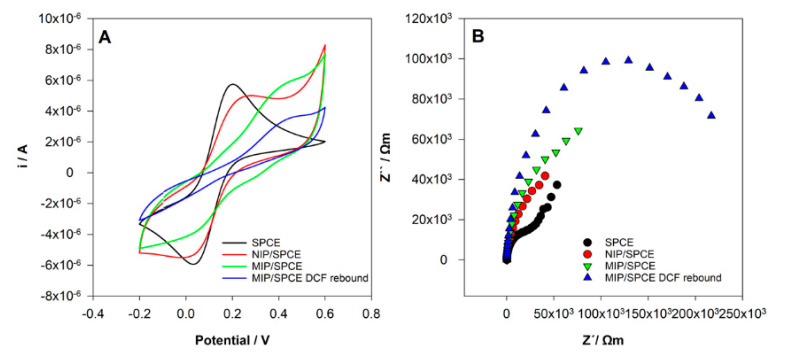
Sensor’s construction characterization. (**A**) Cyclic voltammetry (CV) voltammograms and (**B**) electrochemical impedance spectroscopy (EIS) Nyquist diagrams in 0.5 mM [Fe(CN)_6_]^3−/4−^ in 0.1 M KCl.

In CV analysis (Figure 6A), it can be seen two well-defined redox peaks for the unmodified SPCE as expected. After NIP and MIP preparation, significant differences from SPCE were observed with a decrease in the redox peaks current. This behavior shows that the polymer films were obtained, and the surface was successfully modified. A clear difference between NIP and MIP films was also registered. In the MIP film, the oxidation peak of [Fe(CN)_6_]^3−/4−^ was moved to higher potential and the redox peak was not well defined. These results may be justified by the formation of a polymer with area, conductivity and electroactivity to the redox probe very different when DCF was present during the polymerization. Hence, obviously, polymers with distinctive characteristics were obtained. Finally, after incubation at MIP/SPCE, it was registered a decrease in the electrochemical response showing the rebind of DCF molecules at the surface of the electrode.

The EIS results show similar behavior (Figure 6B). The small semicircle of the Nyquist plot at SPCE corresponds to a fast electron transfer kinetics. After NIP and MIP film formation, there is an increase of impedance with differences between them. This indicates that the surface was more resistant to electron transfer due to the polymer’s formation. After the incubation of DCF at MIP/SPCE, a pronounced increase in the semicircle occurred, demonstrating that DCF molecules were successfully bound to the sensor.

### 3.5. Analytical Performance

**Figure 7:**
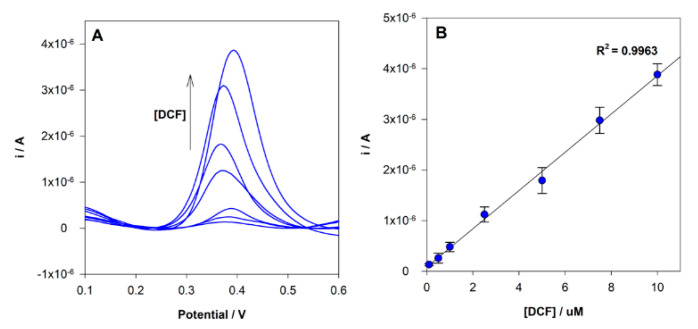
(**A**) DPV voltammograms obtained for MIP sensor analysis of different DCF concentrations; (**B**) linear relationship between peak current intensity and DCF in the concentration range 0.1 to 10.0 µM.

Under the optimized experimental conditions described above, the analysis of DCF at MIP/SPCE using different concentrations in the range between 0.1 to 10 µM was performed by DPV (Figure 7). A linear relationship between the concentration and the peak current was found in the range tested, and the corresponding calibration curve had the following analytical parameters a *r*^2^ = 0.9963, ip(A) = 3.78 × 10^−7^ × [DCF](μM) + 8.50 × 10^−8^. The limit of detection (LOD) and quantification (LOQ) were estimated to be 70 nM and 200 nM, respectively, given by equations: LOD = 3 s/m and LOQ = 10 s/m, where “s” is the standard deviation of the intercept and “m” is the slope of the calibration plot. Each sensor was reused 3 times without a significant change in the current response.

The LOD of the proposed sensor is lower than the reported by other MIPs electrochemical sensors, 100 nM [40] and 1.1 mg/L (3.5 µM) [41]. The method’s precision was evaluated by both intra-day and inter-day repeatability. Four analyses per day during three days were performed. The intra-day repeatability, expressed as relative standard deviation (RSD), was estimated to be 7.5%, and the inter-day repeatability was 11.5% (both evaluated by measuring a solution of 10μM).

### 3.6. Selectivity Studies

The study of the selectivity of the prepared MIP/SPCE was performed by comparison with other drug molecules, mefenamic acid (MFA), carbamazepine (CBZ), acetylsalicylic acid (ASA) and amitriptyline (AMI). MIPs and NIPs sensors were prepared, and solutions of each compound (using the same concentration) were analyzed individually.

**Figure 8:**
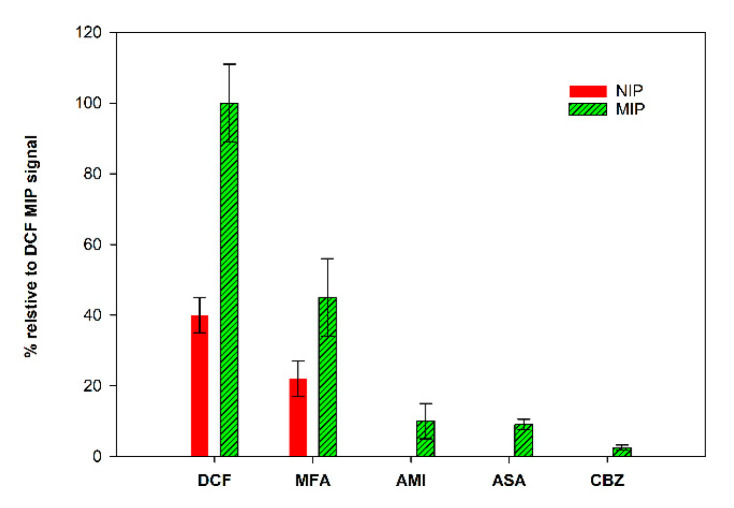
Selectivity studies.

The obtained results, expressed as % of the peak current relative to the MIP peak current obtained for DCF, are summarized in Figure 8. It is possible to conclude that the sensor was quite selective. These studies were performed with analog molecules, both in size and in terms of functional groups. The response for CBZ, ASA and AMI was very low, indicating that the prepared sensor can discriminate these molecules. The higher response was found to MFA, the molecule with more similarities to DCF. Even though the signal for DCF was significantly higher, these results demonstrate that the binding sites of the constructed sensor show special recognition ability toward DCF molecules.

### 3.7. Application to Real Samples

Tap water and water collected from the Lis River (Portugal) were used to test the sensor´s applicability. Both samples were spiked with two different DCF concentrations (2 and 6 µM). The analysis was executed by incubating the samples directly on the MIP sensor without any pretreatment step. The quantification was achieved by the standard addition method. The results are summarized in Table 1. The MIP sensor was successfully able to determine the DCF concentration in the tested samples. Recoveries between 88 and 115% were found with RSD between 3 and 7%, which are good analytical results for this type of sensor. The results proved that the developed MIP sensor could be used as a green, low-cost, easy to handle and fast method for DCF determination.

## 4. Conclusions

A simple, low-cost and disposable MIP electrochemical sensor for the selective determination of DCF was successfully constructed. By using electropolymerization, a simple and efficient preparation methodology, a poly(dopamine) film was obtained from the monomer dopamine with specific binding sites to which DCF can access. The formation of the polymer was characterized by CV and EIS. The sensor showed good analytical performance and recognition selectivity in comparison with other drugs with similar structures and sizes. Furthermore, it was applied in the determination of DCF in real water samples with good analytical parameters. The proposed sensor and preparation methodology showed potential for mass production and to be customized for commercial applications.

## Figures and Tables

**Table 1:** Determination of DCF concentration in spiked water samples with the constructed MIP sensor.

Sample	[DCF]add/µM	[DCF]det/µM	Recovery/%	RSD/%
Tap water	0	-	-	-
	2	1.76	88	7
	6	5.50	92	4
River Water	0	-	-	-
	2	2.30	115	7
	6	6.48	108	3

[DCF]add: DCF concentration added to the sample; [DCF]det: DCF concentration determined in the sample.

## Data Availability

Not applicable.
